# Case Reports of TFE3-Rearranged Renal Cell Carcinoma: FDG-PET Uptake Might Help Diagnosis

**DOI:** 10.15586/jkcvhl.v10i3.266

**Published:** 2023-09-27

**Authors:** Sho Murakami, Keita Nagawa, Takanori Inui, Aya Yamamoto, Mizuka Suzuki, Fumitaka Koga, Toru Motoi, Yasunobu Takaki

**Affiliations:** 1 Department of Radiology, Tokyo Metropolitan Cancer and Infectious Diseases Center Komagome Hospital, Tokyo, Japan;; 2 Department of Urology, Tokyo Metropolitan Cancer and Infectious Diseases Center Komagome Hospital, Tokyo, Japan;; 3 Department of Pathology, Tokyo Metropolitan Cancer and Infectious Diseases Center Komagome Hospital, Tokyo, Japan

**Keywords:** case reports, FDG PET-CT, SUVmax, TFE3-rearranged, Xp11.2 translocation

## Abstract

Translocation and transcription factor E3 (TFE3)-rearranged renal cell carcinoma (RCC) is a rare subtype of RCCs characterised by the fusion of the TFE3 transcription factor genes on chromosome Xp11.2 with one of the multiple genes. TFE3-rearranged RCC occurs mainly in children and adolescents, although middle-aged cases are also observed. As computed tomography (CT)/magnetic resonance imaging (MRI) findings of TFE3-rearranged RCC overlap with those of other RCCs, differential diagnosis is often challenging. In the present case reports, we highlighted the features of the fluorine-18-labelled fluorodeoxyglucose positron emission tomography with CT (FDG PET-CT) in TFE3-rearranged RCCs. Due to the rarity of the disease, FDG PET-CT features of TFE3-rearranged RCC have not yet been reported. In our cases, FDG PET-CT showed high standardised uptake values (SUVmax) of 7.14 and 6.25 for primary tumours. This might imply that TFE3-rearranged RCC has high malignant potential. This is conceivable when the molecular background of the disease is considered in terms of glucose metabolism. Our cases suggest that a high SUVmax of the primary tumour is a clinical characteristic of TFE3-rearranged RCCs.

## Introduction

Translocation and transcription factor E3 (TFE3)-rearranged renal cell carcinoma (RCC) is characterised by the fusion of the TFE3 transcription factor genes on chromosome Xp11.2 with one of the multiple genes. This type of RCC was first classified by the World Health Organization (WHO) in 2004 and is called Xp11.2 translocation RCC (1). In the 2021 WHO classification, it was renamed TFE3-rearranged RCC (2).

This type of RCC is generally diagnosed in paediatric RCCs, and cases of TFE3-rearranged RCC in adults are rare (3). It is difficult to distinguish TFE3-rearranged RCC from other types of RCCs such as clear cell, papillary, chromophobe RCC, oncocytoma and malignant lymphoma on routine imaging studies alone.

In this case series, we report two cases of TFE3-rearranged RCC in young to middle-aged patients. We discuss the imaging features of each case, including computed tomography (CT), magnetic resonance imaging (MRI) and fluorine-18-labelled FDG PET with CT (FDG PET-CT). Finally, we reviewed the typical imaging features of TFE3-rearranged RCC and highlighted the importance of FDG PET-CT uptake.

## Case Report

### Case 1

**Figure 1:**
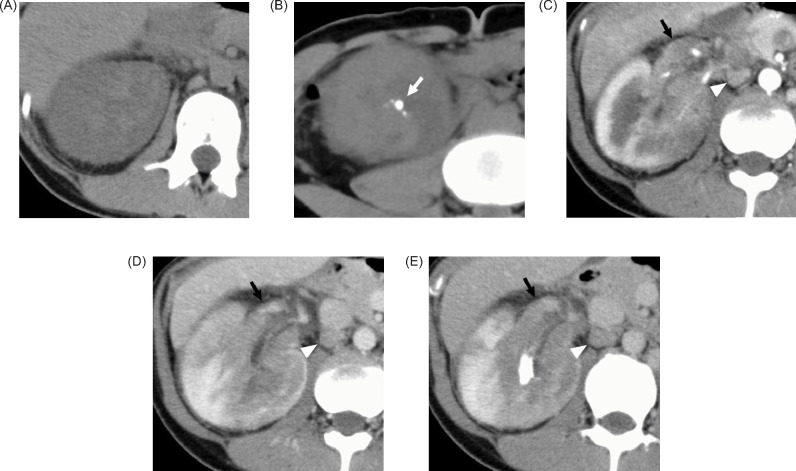
CT revealed the mass in the right kidney with a right renal vein thrombosis (black arrows). At non-enhanced CT (A, B), the mass was slightly hyperattenuating compared with the adjacent cortex with calcifications on the caudal side of the mass (white arrow). At dynamic contrast-enhanced CT (C, D, E), the solid portions of the mass were enhanced less intensely compared with the adjacent renal cortex and enhanced more intensely compared with the adjacent renal medulla during the corticomedullary (C) and nephrographic phase (D) of enhancement. The mass showed slight washout during excretory phase (E). There was lymphadenopathy approximately 14 × 8 mm in the right renal hilus (white arrowheads), and metastasis was suspected.

**Figure 2:**
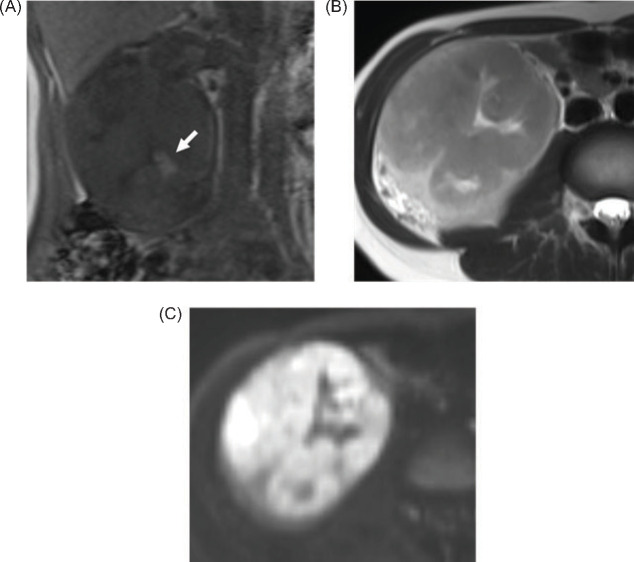
At T1-weighted magnetic resonance imaging (MRI) in coronal (A), the right renal mass was slightly hyperintense compared with the renal cortex, with scattered high-intensity areas that appeared to be haemorrhage (arrow). At half-Fourier acquisition single-shot turbo spin-echo (HASTE) T2-weighted imaging (B), the mass has relatively uniform low intensity with a slight mixture of hyperintense areas. The mass appeared to be hyperintense on diffusion-weighted magnetic resonance imaging (MRI) (C).

**Figure 3:**
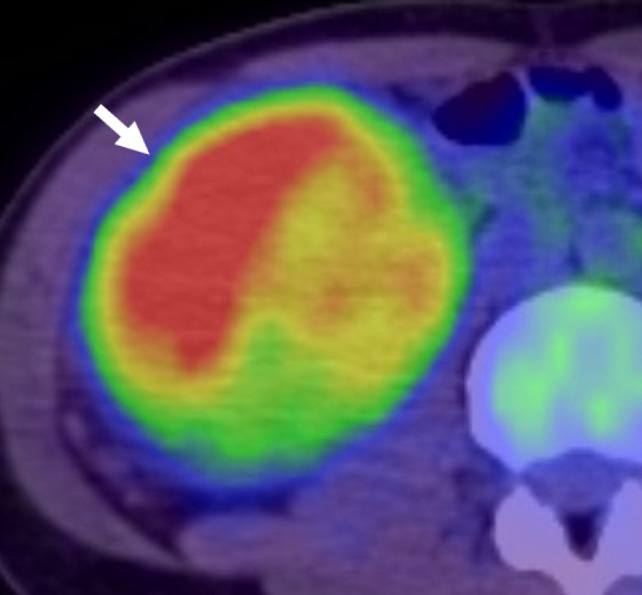
18F-FDG PET-CT images show that the standardised uptake value (SUVmax) of the right renal mass was 7.14 (arrow).

A 29-year-old female patient visited her previous doctor with a chief complaint of right back pain. CT revealed a right renal mass, and a malignant renal tumour was suspected. The patient was referred to our urology department. On plain CT, the tumour was approximately 10 cm in size in the right kidney, with calcifications in the caudal portion (Figure 1A–B). Contrast-enhanced studies revealed tumour thrombus extension into the right renal vein (Figure 1C–E) and lymphadenopathy in the right renal hilus (Figure 1C–E). She had multiple tiny pulmonary masses, suggestive of pulmonary metastases. On T1 and T2-weighted imaging, the tumour was slightly heterogeneous and had an abnormal intensity area that was suspicious for haemorrhage (Figure 2A–B). The mass appeared hyperintense on diffusion-weighted MRI (Figure 2C). FDG PET-CT, which was used for a staging purpose, revealed mild FDG uptake in renal hilar lymphadenopathy and tumour thrombus extending into the right renal vein, and no increased FDG uptake in pulmonary masses. Notably, the tumour showed partially increased FDG uptake, with a standardised uptake value (SUVmax) of 7.14 (Figure 3).

The patient underwent CT-guided biopsy. Histologically, tumour cells with rounded nuclei and slightly eosinophilic or clear cytoplasm were arranged in solid nests with occasional cystic or tubular patterns. The tumour cells were positive for PAX8, cytokeratin AE1/AE3, CD10 and TFE3, and negative for CK7.

We proposed radical nephrectomy with tumour thrombectomy and lymphadenectomy for locally advanced TFE3-rearranged RCC. As we considered that her surgery should be supported by vascular surgeons who were absent in our cancer centre, she was referred to an appropriate facility. She underwent cytoreductive nephrectomy with tumour thrombectomy and lymphadenectomy at our hospital. The pathological diagnosis was TFE3-rearranged RCC, pT3a, pN2 (five positive nodes out of nine removed lymph nodes), v1, ly1 and negative surgical margins. TFE3 immunohistochemistry revealed diffuse positive nuclear staining. The patient developed pulmonary and bony metastases. Despite sunitinib and second-line nivolumab, 9 months after surgery, the patient succumbed to the disease.

### Case 2

**Figure 4:**
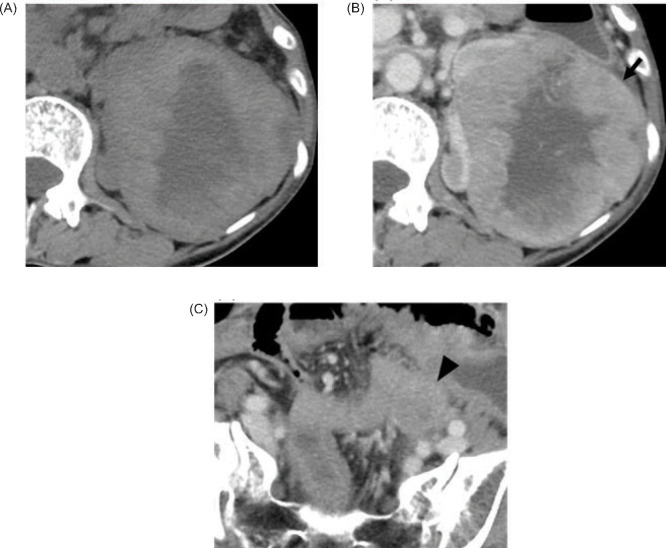
At non-enhanced CT (A), the margins of the mass on the left kidney were hyperattenuating compared with the renal parenchyma, and the interior portion of the mass was hypoattenuating. At contrast-enhanced CT (B, C), the solid portions enhanced less intensely compared with the adjacent renal cortex and enhanced more intensely compared with the adjacent renal medulla. The interior portion of the mass was poorly contrasted, and necrosis was suspected. A capsule-like structure was observed at the tumour margins (arrow). There was lymphadenopathy approximately 25 × 25 mm in the left common iliac area (arrowhead), and metastasis is suspected.

**Figure 5:**
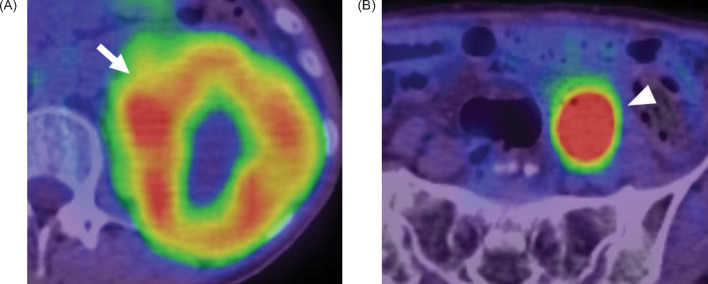
18F-FDG PET-CT images (A, B) show that the SUVmax of the mass on the left kidney was 6.25 (arrow). SUVmax of the lymphadenopathy in the left common iliac area was 3.71 (arrowhead), and metastasis was suspected.

During follow-up after chemoradiotherapy for cervical cancer, a 56-year-old female patient was found to have elevated serum CA125 levels (76.4 U/mL). CT revealed a mass approximately 8.5 cm in size in the left kidney (Figure 4A–B) and lymphadenopathy in the left common iliac area (Figure 4C). FDG PET-CT imaging, performed for staging purposes, showed increased FDG uptake focally in the renal mass and prominently in the lymphadenopathy with respective SUVmax of 6.25 and 7.31 (Figure 5).

The patient was started on pazopanib for metastatic RCC in a presurgical setting. Since her tumours slightly increased in size, she received axitinib as a second-line treatment. Two months later, both the primary tumour and lymphadenopathy shrunk by 14 and 20% in diameter, respectively. Surgical excision of the tumour was planned. She underwent nephrectomy but not lymphadenectomy because the lymph node disease had invaded the sigmoid colon, which was pale and contracted due to radiation-induced colitis. An attending colorectal surgeon did not recommend *en bloc* resection of the sigmoid colon or lymphadenopathy because of the serious risk of anastomotic failure due to radiation-induced colitis.

**Figure 6:**
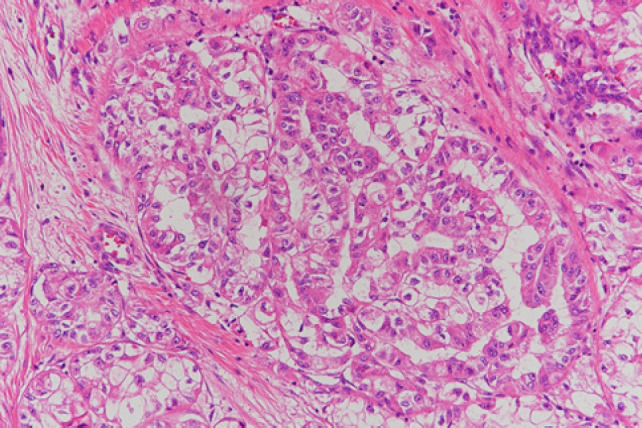
Eosin-stained section of renal tumour: tumour cells with slightly eosinophilic cytoplasm and round nuclei are forming irregular tubular or papillary structure.

**Figure 7:**
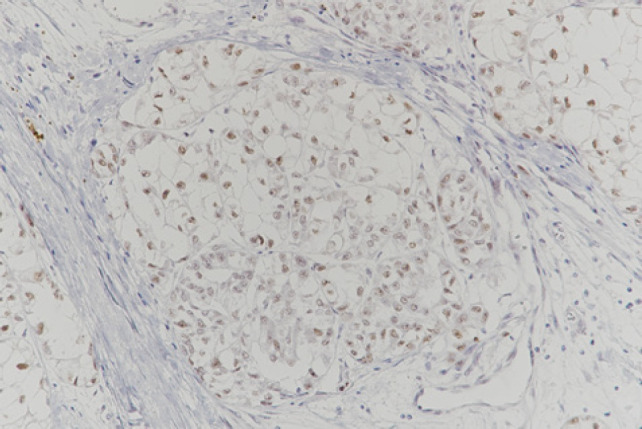
TFE-stained section of renal tumour. The nuclei of tumour cells are diffusely positive for TFE3.

Macroscopically, an encapsulated yellowish solid tumour measuring 8 cm in maximum diameter showed an exophytic growth pattern. Histologically, the tumour cells were arranged in a solid, alveolar or tubular pattern with extensive necrosis. As the cytoplasm of most tumour cells was clear or slightly eosinophilic, it resembled clear-cell carcinoma (Figure 6); however, the tumour cells were positive for epithelial membrane antigen (EMA) (focal), CD10 and TFE3 (Figure 7), and negative for CK7. Collectively, the tumour was diagnosed as a TFE3-rearranged RCC.

Although systemic therapy was switched to nivolumab, the patient developed metastases to the left oblique abdominal muscles, which were surgically resected 4 months after nephrectomy. Nine months later, the patient developed local recurrence in the left renal fossa and *de novo* invasive sigmoid colon cancer. She underwent resection of the local recurrence, *en bloc* resection of the sigmoid colon and persistent common iliac lymphadenopathy. Finally, the patient achieved complete surgical remission and discontinued nivolumab therapy. Since the last surgery, the patient has been disease-free for 3.5 years (4.5 years have passed since cytoreductive nephrectomy).

## Discussion

RCC with fusions of the TFE3 transcription factor genes on chromosome Xp11.2 with one of the multiple genes is a rare and independent subtype of RCC with high malignant potential. This type of RCC is called Xp11.2 translocation renal carcinoma, but in the 2021 WHO classification, it was renamed TFE3-rearranged RCC (2). In 2016, this type of RCC was included in the new category of MiT (microphthalmia-associated transcription factor) family translocation RCC (4). The origin of this disease reflects the fact that it is characterised by fusions involving the *TFE3* gene on chromosome Xp11.2, leading to overexpression of the TFE3 protein in the nucleus of cancer cells (5).

TFE3-rearranged RCC is generally a paediatric RCC, accounting for 40% of all cases, whereas adult RCCs account only for 1.6–4% (2). In particular, it was reported that only seven cases were observed in patients aged >65 years (3). Studies have demonstrated that the prognosis in children is better than that in adults (5, 6). However, as shown in a recent systematic review and meta-analysis, no significant differences were observed in the prognosis between children and adults and between females and males (7). Thus, the dispute over whether age and sex have an impact on the prognosis of tumours continues to date.

TFE3-rearranged RCC is a type of RCC with a relatively low incidence and various prognoses. Early stage TFE3-rearranged RCC has a similar prognosis to most typical RCCs, but late-stage TFE3-rearranged RCC can lead to poor oncological outcomes (8).

Similar to other types of RCCs, the presence of symptoms and advanced disease stage, particularly the presence of lymph node and distant metastasis, are reportedly associated with worse prognosis in patients with TFE3-rearranged RCCs (8). In this respect, Case 1, in which right back pain and metastasis to the lung, bone and lymph nodes were found at presentation, showed poor prognosis. Although lymph node metastasis was suspected in Case 2, she was asymptomatic at diagnosis and finally achieved complete surgical remission in combination with systemic therapy.

On gross specimen inspection, TFE3-rearranged RCCs appear as variegated tan-yellow or brownish masses with necrotic and haemorrhagic areas (9). Histologically, the most distinctive morphological pattern is the presence of papillary architecture composed of epithelioid clear cells. However, different architectures have been reported, such as solid, nested, trabecular and microcystic pattern (4). TFE3 immunostaining, initially considered the most sensitive and specific marker, should be cautiously used because of the infrequent false-positive and false-negative results (4). The essential diagnostic criteria are the identification of strong unclear labelling for TFE3 by immunohistochemistry in a clean background, TFE3 rearrangement identified by break-apart FISH or TFE3 gene fusion identified by RNA sequencing (2).

On imaging, TFE3-rearranged RCCs generally have a heterogeneous appearance owing to the solid and cystic components with haemorrhage, necrosis and calcifications (9). In non-enhanced CT, TFE3-rearranged RCC is usually hyperattenuating (45–60 Hounsfield Unit: HU) compared with the adjacent cortex (9). Focal or rim-like calcifications are noted on CT images in 23.8–60.0% of cases (9). On contrast-enhanced CT, the solid portions typically enhance mildly to moderately and less intensely compared with the adjacent cortex during all three phases of enhancement and enhance more intensely compared with the adjacent medulla, except during the delayed phase (9). Prolonged enhancement in delayed-phase CT is also typical (9). The tumour margins are well defined, and a progressively enhancing peripheral rim is common (76.2% of cases) and best seen on delayed-phase CT images (9). On T2-weighted MRI, it is most often heterogeneous in signal intensity, regardless of its size (9). The signal intensity of TFE3-rearranged RCC on T1-weighted MRI is often iso- to slightly hyperintense compared to in the cortex (9). The mass appeared slightly hyperintense on diffusion-weighted MRI (*b* = 800 s/mm^2^).

Representative differential diagnosis of TFE3-rearranged RCC could include other types of RCCs, such as clear cell, papillary or chromophobe RCC, malignant lymphoma, renal pelvis carcinoma and collecting duct carcinoma. As described in some reports, it is often difficult to distinguish between them using imaging alone. Papillary RCC tends to resemble TFE3-rearranged RCC, showing a hypovascular tumour in the corticomedullary phase after contrast injection (10). In our cases, we could raise the possibility of TFE3-rearranged RCC in Case 1 because of a young adult case and the image characteristics consistent with TFE3-rearranged RCC, although the image characteristics were also similar to those of papillary RCC. Case 2 was a hypovascular tumour with intralesional cystic changes or necrosis, a capsule-like structure and lymph node metastasis, which was consistent with the imaging features of TFE3-rearranged RCC. However, because the patients were middle-aged or older, it was necessary to consider other malignancies, including papillary RCC, chromophobe RCC, renal pelvic carcinoma and collecting duct carcinoma.

As an essential imaging modality in oncological practice, FDG PET-CT is used to detect viable tumour tissue and evaluate disease activity. However, probably because of the rarity of the disease, FDG PET-CT features of TFE3-rearranged RCC have not been reported to date. For the detection of primary RCCs, a review by Wang et al. reported that FDG PET-CT had a sensitivity of only 62% (11). Thus, the role of FDG PET-CT seems to be limited in the evaluation of primary RCCs. However, the histological grade of renal tumours might be correlated with FDG uptake, and the SUVmax of high-grade tumours is significantly higher than that of low-grade tumours among clear cell and papillary RCCs (12, 13). The SUVmax of low-grade clear-cell RCCs was 2.3 ± 0.6, while that of high-grade diseases was 6.2 ± 4.9 (14). It was reported that an optimal SUV cut-off value of 3.0 had a sensitivity of 89% and specificity of 87% in differentiating between high-grade and low-grade clear-cell RCCs, and that the SUVmax of high-grade (WHO grade 3 and 4) papillary RCCs was 9.44 ± 6.18 (12, 13).

In our two cases, the SUVmax of TFE3-rearranged RCCs were 7.14 and 6.25, respectively. This value was higher than the SUVmax of low-grade clear-cell carcinoma and comparable to that of high-grade primary RCCs. This might imply that TFE3-rearranged RCC has high malignant potential.

At the molecular level, FDG uptake depends on the first steps of the glucose metabolism pathway, which includes components such as glucose transporters (GLUTs), hexokinase and glucose-6 phosphatase. GLUT-1 is the most important component for FDG accumulation in various tumours, but immunohistochemical studies have shown that subtypes such as GLUT-5 have stronger staining in clear-cell RCC than that observed in other subtypes (15, 16). c-MET/HGF signalling is known to activate glucose metabolism by enhancing the expression of GLUTs (17). In a systematic study assessing the molecular status of c-MET in clear-cell RCC, high c-MET expression and copy number were associated with high-grade carcinoma and metastatic disease (18). Moreover, in specific subtypes of TFE3-rearranged RCCs, aberrant activation of the c-MET/HGF signalling cascade induced by oncogenic TFE3 fusion proteins has been reported. Strong c-MET upregulation was observed in ASPL-TFE3 gene fusions, whereas moderate activation was also observed in PSF-TFE3 and NONO-TFE3 fusions as well (14, 19). These observations might explain the propensity of FDG uptake described above: TFE3-rearranged RCC and high-grade clear-cell RCC show a more aggressive nature, higher activation of glucose metabolism by c-MET/HGF signalling and hence higher FDG uptake compared to low-grade clear-cell RCC.

Based on the results of these previous studies and ours, TFE3-rearranged RCC is expected to have a high SUVmax, comparable to that of high-grade RCC. FDG PET-CT is not an imaging modality that is commonly used in RCC, but it might have potential benefits for the diagnosis and staging of TFE3-rearranged RCC considering the above description. Thus, we consider FDG PET-CT as an adjunctive test especially in young adult cases and those in which renal tumours show aggressive features on CT/MRI and are difficult to diagnose with conventional RCCs. With future data accumulation, FDG PET-CT, together with clinical information and CT/MRI findings, may be useful in differentiating TFE3-rearranged RCC from other renal tumours.

## Conclusion

The CT and MRI findings of TFE3-rearranged RCC tend to be similar to those of other renal carcinomas, and it is often difficult to distinguish them on imaging alone. Due to the rarity of the disease, FDG PET-CT features of TFE3-rearranged RCCs have not been reported to date. Our two cases showed SUVmax comparable to that of high-grade RCC. This might imply that TFE3-rearranged RCC has high malignant potential. Although further case studies are needed, FDG PET-CT in combination with clinical information and CT/MRI findings might help differentiate TFE3-rearranged RCCs from other renal tumours.
